# Effect of ingested human antibodies induced by RTS, S/AS01 malaria vaccination in children on *Plasmodium falciparum* oocyst formation and sporogony in mosquitoes

**DOI:** 10.1186/1475-2875-13-263

**Published:** 2014-07-09

**Authors:** Kazutoyo Miura, Erik Jongert, Bingbing Deng, Luwen Zhou, John P Lusingu, Chris J Drakeley, Michael P Fay, Carole A Long, Johan Vekemans

**Affiliations:** 1 Laboratory of Malaria and Vector Research, National Institute of Allergy and Infectious Diseases, National Institutes of Health, 12735 Twinbrook Parkway, Rockville, MD 20852, USA; 2 GlaxoSmithKline Vaccines, Wavre, Belgium; 3 National Institute for Medical Research, Tanga Centre, Korogwe site, Tanga, Tanzania; 4 Center for Medical Parasitology, Department of Medical Microbiology and Immunology, University of Copenhagen, Copenhagen, Denmark; 5 Faculty of Infectious and Tropical Diseases, London School of Hygiene and Tropical Medicine, London, UK; 6 Biostatistics Research Branch, National Institute of Allergy and Infectious Diseases, National Institutes of Health, Bethesda, USA

**Keywords:** RTS,S/AS01, Oocyst formation, Sporogony, Standard membrane feeding assay

## Abstract

**Background:**

The circumsporozoite protein (CS protein) on the malaria parasites in mosquitoes plays an important role in sporogony in mosquitoes. The RTS,S/AS01 malaria vaccine candidate, which has shown significant efficacy against clinical malaria in a large Phase 3 trial, targets the *Plasmodium falciparum* CS protein, but the ability of serum from vaccinated individuals to inhibit sporogony in mosquitoes has not been evaluated.

**Methods:**

Previously a double-blind, randomized trial of RTS,S/AS01 vaccine, as compared with rabies vaccine, in five- to 17-month old children in Tanzania was conducted. In this study, polyclonal human antibodies were purified from the pools of sera taken one month after the third vaccination. IgGs were purified from four pools of sera from 25 RTS,S/AS01 vaccinated children each, and two pools of sera from 25 children vaccinated with rabies vaccine each. The ability of antibodies to inhibit *P. falciparum* oocyst formation and/or sporogony in the mosquito host was evaluated by a standard membrane-feeding assay. The test antibodies were fed on day 0 (at the same time as the gametocyte feed), or on days 3 or 6 (serial-feed experiments). The oocyst and sporozoite counts were performed on days 8 and 16, respectively. In addition, two human anti-CS monoclonal antibodies (mAb) and a control mAb were also evaluated.

**Results:**

Polyclonal anti-CS IgG preparations from RTS,S-vaccinated children tested at concentrations of 149-210 ELISA units (EU)/ml did not show significant inhibition in oocyst and sporozoite formation when the antibodies were fed with gametocytes at the same time, or later (serial-feed experiments). Similarly, anti-CS mAbs tested at 6,421 or 7,122 EU/ml did not show reduction in oocyst and sporozoite formation.

**Conclusions:**

This study does not support the concept that anti-CS antibodies induced by the RTS,S/AS01 vaccines in humans noticeably reduce malaria transmission by blocking *P. falciparum* sporozoite development or salivary gland invasion in mosquitoes when taken up during feeding.

## Background

There are two ways malaria vaccines could prevent transmission of malaria from one immunized individual to another susceptible individual. One is through the induction of pre-erythrocytic or blood-stage immunity that prevent (or dramatically reduces) gametocyte formation in humans, and the other one is through the induction of immunity that acts in the mosquito to prevent parasites from reaching the salivary glands. The latter strategy relies on the activity of immune effectors ingested with the blood meal against parasite and mosquito antigens that are exposed to the blood meal [1-4].

The RTS,S/AS01 malaria candidate vaccine is being developed with the aim of reducing the clinical disease associated with *Plasmodium falciparum* malaria in children in Africa when administered to infants and/or young children. While the vaccine has shown a significant efficacy with respect to clinical malaria in a Phase 3 trial [5], the ability of RTS,S/AS01 to reduce malaria transmission has not been evaluated. The RTS,S/AS01 vaccine target antigen is the circumsporozoite protein (CS protein), a 412 amino acids protein abundantly associated with the sporozoite surface. CS protein, the expression of which starts in the oocyst [6-8], plays an important role in sporogony [9,10].

Modelling studies have been conducted, and clinical studies are being considered, to evaluate the potential for RTS,S/AS01, if used in mass vaccination programmes achieving high population coverage, to reduce transmission of malaria through the ability of pre-erythrocytic immunity to reduce incidence of new infection [11]. In contrast, this study evaluates the potential for serum from RTS,S/AS01 immunized children, when ingested by the mosquito with a blood meal, to inhibit sporogony in mosquitoes. The rationale for testing this hypothesis comes from two observations. First, it is known that antibodies ingested by the mosquito during a blood meal can traverse the midgut epithelium and reach the haemolymph [12]. Secondly, it has been demonstrated that mosquitoes infected with transgenic fungi which expressed single light chain anti-CS antibody showed fewer sporozoites in the salivary glands compared to the mosquitoes infected with the wild type fungi [13]. In addition, a recent study indirectly supports the idea of transmission blocking by an anti-CS antibody: antibodies against circumsporozoite protein-binding protein (CSPBP) significantly reduced the sporozoite load in salivary glands of *Plasmodium berghei* infected mosquitoes [14]. It is, therefore, possible that anti-CS antibodies induced by RTS,S/AS01 vaccination and ingested by the mosquito during a blood meal may affect oocyst formation and/or sporogony in the mosquito host. Past attempts at evaluating the effect of anti-sporozoite sera on sporogony have led to conflicting results [12,15-18]. This is the first study to evaluate the effect of serum samples from children who were immunized with a vaccine against CS protein.

In this study, the ability of antibodies from children vaccinated with RTS,S/AS01 to inhibit oocyst formation and/or sporogony in the mosquito host was tested using a standard membrane-feeding assay (SMFA). The post-vaccination sera collected in a double-blind, randomized trial of RTS,S/AS01 vaccine as compared with rabies vaccine in five- to 17-month old children in Korogwe, Tanzania were used. The clinical study showed that RTS,S/AS01 provided a 53% (95% confidence interval (CI), 28 to 69; P < 0.001) protection against malaria over an average eight-month period [19]. In the current study, *in vitro* cultured *P. falciparum* gametocytes were mixed with test antibodies and fed to laboratory-reared *Anopheles* mosquitoes. After a few days, mosquitoes were dissected and successful progression of the parasite cycle was evaluated by counting oocysts on the midgut basal lamina, or sporozoites in the salivary glands, depending on the numbers of days passed after the infectious feeding. In addition, serial feedings were used, where mosquitoes were first fed on gametocytes without antibodies and then later fed on test antibodies. These serial feedings mimic the fact that in nature mosquitoes feed repeatedly. In addition, human monoclonal antibodies (mAb) against CS [20] were also evaluated.

## Methods

### Clinical trial and antibody sample preparation

A Phase 2b clinical trial of RTS,S/AS01 vaccine (GlaxoSmithKline (GSK) Biologicals, Belgium) was conducted in children in Korogwe, Tanzania, as part of a multicentre, observer-blinded, randomized, controlled trial reported previously [19]. The study was prospectively registered at ClinicalTrials.gov (NCT00380393) and approved by the Tanzanian Medical Research Coordinating Committee, and the Western Institutional Review Board in Seattle. The study was overseen by an Independent Data Monitoring Committee and local safety monitors, and conducted in accordance with the Helsinki Declaration of 1964 (revised 1996) and Good Clinical Practice guidelines. Written informed consent was obtained from the parent or guardian of each child. In brief, children between five and 17 months of age at the time of first vaccination received either RTS,S/AS01 vaccine or a control rabies vaccine, intramuscularly at zero, one and two months.

Because of the small volume of sera available from each child, the current study was based on six serum pools. Each pool was made using sera from 25 vaccinated subjects bled at one month post-third vaccine dose. Four pools were made from children who received RTS,S/AS01 vaccines, and the other two pools were from rabies-immunized children.

The human anti-RTS, S monoclonal antibodies targeting the repeat region of the CS protein (mAb, MAL1C and MAL2A) were generated as described previously [20]. As a control, a malaria-unrelated human mAb was used for each assay.

### Anti-CS ELISA

Anti-CS IgG concentration in each sample was measured using a validated anti-CS ELISA as described previously [20]. The anti-CS IgG concentration is expressed as ELISA units per milliliter (EU/ml).

### IgG purification and standard membrane-feeding assay (SMFA)

IgG from pooled serum samples were purified via Protein G affinity chromatography (Pierce, Rockford, IL, USA) according to the manufacturer’s instructions and adjusted to a final concentration of 20 mg/ml in phosphate buffered saline (PBS). Four total IgGs from the RTS,S/AS01 group are designated as CS-1, -2, -3 and -4, and two IgGs from the control (rabies) group are designated as Contl-1 and -2 in this study.

The standardized methodology for performing the SMFA has been described previously [21]. Briefly, 16-18 day old gametocyte cultures of the *P. falciparum* NF54 line (200 μl of 50% haematocrit culture adjusted to 0.15-0.2% stage V gametocytaemia) were mixed with 60 μl of a test sample, and the final mixture was immediately fed to ~50 female *Anopheles stephensi* (Nijmegen strain, three to six days old) mosquitoes through a membrane-feeding apparatus. The human serum and red blood cells used for the cultures were purchased from Interstate Blood Bank, Inc, Memphis, TN, USA. The polyclonal IgGs were tested at 3.75 mg/ml of total IgG concentration. For the mAb, the concentration of 0.231 mg/ml was used. The level of anti-CS specific antibody of each sample (either polyclonal or monoclonal antibodies) in a test feeder was calculated based on EU/ml value of the stock IgG and the dilution factor to make the final mixture for a feeding experiment. For example, CS-1 sample showed 805 EU/ml in the stock IgG (at 20 mg/ml of total IgG), and the anti-CS IgG level was calculated as 151 EU/ml in a test feeder (at 3.75 mg/ml of total IgG). Mosquitoes were kept for eight days and dissected (20 mosquitoes per sample) to enumerate oocysts in their midguts. Only midguts from mosquitoes with any eggs at the time of dissection were analysed.

For the sporozoite count, mosquitoes were kept for 16 days and dissected to collect salivary glands (12 mosquitoes per sample). The salivary glands from an individual mosquito were transferred to a tube containing 50 μl of PBS, then homogenized gently by repeated pipetting. Twenty μl of suspension were transferred to a haemocytometer and the number of sporozoites was determined under a phase-contrast microscope.

For a serial-feed experiment, ~50 female *An. stephensi* were fed gametocyte cultures as in a regular SMFA, but without any test antibody, on day 0 (100 μl infected erythrocytes and 160 μl normal human serum). On days 3 or 6, the mosquitoes were fed again with a test antibody and 50% haematocrit of uninfected erythrocytes (60 μl test antibody diluted in PBS was mixed with 100 μl uninfected erythrocytes and 100 μl normal human serum). The final protein concentration of a test IgG in a feeder was the same as a regular SMFA: 3.75 mg/ml for polyclonal IgGs and 0.231 mg/ml for mAbs. The oocyst and sporozoite counts were performed on days 8 and 16 post-infectious feed, respectively. In these experiments, salivary glands from 12 mosquitoes for each test sample were pooled in 300 μl PBS for counting.

### Statistical analysis

Per cent (%) inhibition of mean oocyst intensity (PIm[o]) was calculated as: 100 × {1 - (mean number of oocysts in the test group)/(mean number of oocysts in the control groups)}. Similarly,% inhibition of mean sporozoite intensity (PIm[s]) was calculated. For the calculations, an arithmetic mean was used, instead of geometric mean or median, because previous work on oocyst intensity has shown a zero inflated negative binomial distribution fits very well to the data [21]. Other studies also utilized similar negative binomial models for the oocyst data [22,23]. For sporozoite intensity, a study has shown that the log sporozoite intensity is approximately linearly related to the log oocyst intensity [24] in the case of *P. falciparum* infection in *Anopheles gambiae* mosquitoes. Therefore, it seems like a reasonable assumption to use arithmetic means for sporozoite analyses as well. The% inhibition of oocyst prevalence (PIp[o]) was evaluated as: 100 × {1- (proportion of mosquitoes with any oocysts in the test group)/(proportion of mosquitoes with any oocyst in the control group)}. The lowest number of sporozoite detected by this method (the haemocytometer measurement described above) was 3 × 10^3^ per mosquito. Therefore, the mosquito with ‘zero’ sporozoites might have some sporozoites (<3 × 10^3^) if they were examined with a more sensitive method. For this reason,% inhibition of sporozoite prevalence was not analysed in this study. For estimating the PIm[s] and its CI, the value of 1.5 × 10^3^ (the half of the minimum non-zero count in the current study) was assigned for a mosquito with ‘zero’ sporozoites.

The CI for PIm[o] of each test sample was calculated using a zero-inflated negative binomial random effects model which was similar to the method described previously [21]. For this study the zero-inflation factor and the random effects standard errors for the container of mosquitoes (COM, viz, a group of mosquitoes which were housed in the same container and were fed the same culture and test antibodies) and feeds were estimated from the previous study [21]. Under the model, new data (in which means were obtained from the current study) were simulated and the middle 95% was used as the CI. For the CI of PIp[o] and PIm[s], it was not possible to use the same model as the historical data were not sufficient (in case of PIp[o]) or no such data were available (PIm[s]). Therefore, the method of Miettinen and Nurminen [25] was used for PIp[o] and the negative binomial method for PIm[s]. Both methods did not account for the random effect for COM and feeds, as there were no data to estimate those effects, and hence the results will tend to be anti-conservative, ie, reject more often than the nominal 0.05 level.

For the polyclonal IgG data, a meta regression model was used to compare PIm[o] values between anti-CS IgGs (four samples) and control IgGs (two samples). The same model was utilized to compare PIm[s] values of the two groups for the regular feed. Since the sporozoite counts were done with pools of salivary glands of 12 mosquitoes per group in the serial-feed experiments, a *t*-test was used to compare the PIm[s] data. The PIp[o] data of two groups were compared by a logistic regression allowing for over-dispersion.

To combine the data from two independent feeding experiments, a normal fixed effects meta analysis method on the log relative risks was used. The metaphor R package was used for the analysis [26].

All statistical tests were performed in R (version 2.15.2) and two-sided p-values <0.05 were considered significant.

## Results

### Transmission-blocking activity of IgGs from human sera of RTS,S-vaccinated children

The purified total IgGs of CS-1, -2, -3 and -4 (at 3.75 mg/ml) corresponded to anti-CS concentrations of 151, 210, 149 and 156 EU/ml, respectively, while control IgGs, Contl-1 and -2, showed less than 0.1 EU/ml. The concentrations of anti-CS antibodies (149-210 EU/ml) were lower than levels observed at peak (one month post dose 3, geometric mean of 539.6 EU/ml [19]), but higher than that observed a few months later (71.9 EU/ml 1.5- to 7.5 (mean of 5) months after the peak) [19], and similar to peak levels in young infants (199.9 EU/ml) [27] and in 1-4 years old children (207 EU/ml) [28].

Table 1 shows% inhibition (and the CI) of mean oocyst intensity (PIm[o]), oocyst prevalence (PIp[o]), and mean sporozoite intensity (PIm[s]) of each anti-CS IgG compared to the Control IgGs. Since there were huge variations in oocyst and sporozoite numbers even within the same COM, the estimates of% inhibition had large CIs (Table 1). Therefore, four anti-CS IgGs and two control IgGs were compared as groups. In terms of the oocyst intensity, the model estimated that the mean in the anti-CS IgG group was 1.0 (95% CI, -2.7 to 4.7; p = 0.59) higher than that in the control. The odds ratio of oocyst prevalence in anti-CS IgG group compared to control was estimated as 2.7 (95% CI, 0.7 to 10.7; p = 0.15). For the sporozoite intensity, the model estimated the mean in anti-CS IgG group was 1.6 × 10^3^ (95% CI, -2.9 to 6.2 × 10^3^; p = 0.48) higher. Taken together, there was no significant inhibition in anti-CS IgGs compared to the control IgGs for any readouts.

**Table 1:** **Transmission-blocking activities of human polyclonal IgGs fed with infected blood meal**^a^

**Sample**	**Mean ooc**^**b**^	**PIm[o]**^**c**^	**p-value**^**d**^	**Mosquito**^**e**^	**PIp[o]**^**f**^	**p-value**^**g**^	**Mean Spz**^**h**^	**PIm[s]**^**i**^	**p-value**^**j**^
CS-1	10.7	4	0.87	1/20	-9	0.36	14.8	-51	0.32
	(0, 34)	(-142, 64)			(-30, 14)		(0, 52.3)	(-257, 31)	
CS-2	10.8	3	0.88	1/20	-9	0.36	15.3	-56	0.29
	(0, 20)	(-134, 65)			(-30, 14)		(0, 43.0)	(-274, 30)	
CS-3	12.2	-9	0.95	1/20	-9	0.36	10.1	-3	0.94
	(0, 38)	(-174, 59)			(-30, 14)		(0, 19.3)	(-152, 54)	
CS-4	14.2	-27	0.67	1/20	-9	0.36	9.1	6	0.89
	(0, 26)	(-208, 53)			(-30, 14)		(0, 41.3)	(-150, 61)	
Contl-1	10.6	N/A	N/A	2/20	N/A	N/A	8.9	N/A	N/A
	(0, 49)						(0, 22.8)		
Contl-2	11.7	N/A	N/A	3/20	N/A	N/A	10.6	N/A	N/A
	(0, 34)						(0, 25.3)		

^a^All purified IgGs were tested at 3.75 mg/ml and fed with infected blood meal at the same time.

^b^Arithmetic mean (range) of oocysts per mosquito midgut.

^c^Per cent inhibition of mean oocyst intensity (PIm[o]) and the 95% confidence interval (95% CI).

^d^Two-sided p-values for testing whether PIm[o] is significantly different from 0.

^e^Number of mosquitoes without any oocysts/number of mosquitoes examined.

^f^Per cent inhibition of prevalence of mosquitoes with oocysts (PIp[o]) and the 95% CI.

^g^p-values of PIp[o].

^h^Arithmetic mean (range) of sporozoites (×10^3^) per mosquito.

^i^Per cent inhibition of mean sporozoite intensity (PIm[s]) and the 95% CI.

^j^p-values of PIm[s].

Since CS protein is only synthesized six days or later after a gametocyte feed in a mosquito [9], a serial-feed experiment, where the same mosquitoes were fed again with test antibodies on days 3 or 6 after gametocyte feed, was performed. The inhibitory activities of each individual test IgG are shown in Table 2 (day 3 serial feed) and Table 3 (day 6 serial feed). Similar to the regular feed (Table 1), the variations in the oocyst numbers were large. Therefore, the effect of anti-CS IgG was also evaluated as a group. For the oocyst intensity, the mean in the anti-CS IgG group was 2.7 (95% CI, -6.2 to 11.6; p = 0.55) or 2.2 (95% CI, -3.9 to 8.3; p = 0.48) higher than those in control in the day 3 or 6 serial feeds, respectively. The odds ratio of oocyst prevalence in the anti-CS IgG group compared to control was 0.32 (95% CI, 0.02 to 6.0; p = 0.49) in the day 3 serial feed. In the day 6 serial feed, since only one mosquito (who was fed with CS-2) with zero oocysts (all other mosquitoes in both groups had more than one oocyst), no comparison was performed for the prevalence data. For the sporozoite numbers, there was no significant difference between the two groups (p = 0.34 for day 3 serial feed, and p = 0.43 for day 6 serial feed).

**Table 2:** **Transmission-blocking activities of human polyclonal IgGs fed three days post-infected blood meal**^a^

**Sample**	**Mean ooc**^**b**^	**PIm[o]**^**c**^	**p-value**^**d**^	**Mosquito**^**e**^	**PIp[o]**^**f**^	**p-value**^**g**^	**Mean Spz**^**h**^	**PIm[s]**^**i**^
CS-1	30.0	1	0.93	3/20	13	0.07	71.3	-184
	(0, 75)	(-148, 62)			(-1, 35)			
CS-2	29.9	1	0.92	0/20	-3	0.48	49.4	-97
	(2, 56)	(-139, 62)			(-15, 14)			
CS-3	37.6	-24	0.66	0/20	-3	0.48	56.9	-127
	(5, 69)	(-190, 51)			(-15, 14)			
CS-4	34.5	-14	0.88	3/20	13	0.07	35.4	-41
	(0, 87)	(-175, 57)			(-1, 35)			
Contl-1	25.5	N/A	N/A	0/20	N/A	N/A	43.1	N/A
	(1, 80)							
Contl-2	35.0	N/A	N/A	1/20	N/A	N/A	7.0	N/A
	(0, 83)							

^a^All purified IgGs were tested at 3.75 mg/ml and fed three days post-infected blood meal.

^b^Arithmetic mean (range) of oocysts per mosquito midgut.

^c^Per cent inhibition of mean oocyst intensity (PIm[o]) and the 95% confidence interval (95% CI).

^d^Two-sided p-values for testing whether PIm[o] is significantly different from 0.

^e^Number of mosquitoes without any oocysts/number of mosquitoes examined.

^f^Per cent inhibition of prevalence of mosquitoes with oocysts (PIp[o]) and the 95% CI.

^g^p-values of PIp[o].

^h^Number of sporozoites (×10^3^) per mosquito calculated from a pool of salivary glands of 12 mosquitoes.

^i^Per cent inhibition of mean sporozoite intensity (PIm[s]) calculated with the average of sporozoites counts of Contl-1 and Contl-2.

**Table 3:** **Transmission-blocking activities of human polyclonal IgGs fed six days post-infected blood meal**^a^

**Sample**	**Mean ooc**^**b**^	**PIm[o]**^**c**^	**p-value**^**d**^	**Mosquito**^**e**^	**PIp[o]**^**f**^	**p-value**^**g**^	**Mean Spz**^**h**^	**PIm[s]**^**i**^
CS-1	37.7	-13	0.82	0/20	0	1.00	91.1	-2
	(19, 82)	(-176, 54)			(-10, 16)			
CS-2	33.7	-1	0.97	1/20	5	0.15	72.7	19
	(0, 54)	(-151, 63)			(-4, 24)			
CS-3	35.8	-8	0.94	0/20	0	1.00	87.4	3
	(5, 72)	(-157, 59)			(-10, 16)			
CS-4	34.6	-4	0.98	0/20	0	1.00	83.8	7
	(6, 74)	(-144, 61)			(-10, 16)			
Contl-1	33.2	N/A	N/A	0/20	N/A	N/A	84.8	N/A
	(10, 62)							
Contl-2	33.3	N/A	N/A	0/20	N/A	N/A	94.7	N/A
	(10, 77)							

^a^All purified IgGs were tested at 3.75 mg/ml and fed six days post-infected blood meal.

^b^Arithmetic mean (range) of oocysts per mosquito midgut.

^c^Per cent inhibition of mean oocyst intensity (PIm[o]) and the 95% confidence interval (95% CI).

^d^Two-sided p-values for testing whether PIm[o] is significantly different from 0.

^e^Number of mosquitoes without any oocysts/number of mosquitoes examined.

^f^Per cent inhibition of prevalence of mosquitoes with oocysts (PIp[o]) and the 95% CI.

^g^p-values of PIp[o].

^h^Number of sporozoites (×10^3^) per mosquito calculated from a pool of salivary glands of 12 mosquitoes.

^i^Per cent inhibition of mean sporozoite intensity (PIm[s]) calculated with the average of sporozoites counts of Contl-1 and Contl-2.

### Transmission-blocking activity of human monoclonal anti-CS antibodies

Two human monoclonal antibodies (MAL1C and MAL2A) were also evaluated in this study. Using the mAb, it was possible to test at higher CS-specific antibody concentration in the membrane-feeding assay. The anti-CS specific antibody levels of MAL1C and MAL2A were 7,122 and 6,421 EU/ml when they were tested at 0.231 mg/ml protein concentration.

In the first experiment with mAb, MAL1C, MAL2A and control (Contl) mAb were fed along with the gametocyte culture. While MAL1C mAb did not show any inhibition, MAL2A showed 61% inhibition (95% CI, -24 to 87; p = 0.089) in oocyst intensity (PIm[o]) and 32% inhibition (95% CI, 7 to 55; p = 0.018) in oocyst prevalence (PIp[o]) in the first feeding experiment (Table 4). A second experiment was performed to confirm this inhibitory activity, but here, MAL2A did not show any inhibition. When the data from the two feeding experiments were combined, MAL2A showed 21% inhibition (95% CI, -73 to 64; p = 0.551) in PIm[o] and 7% inhibition (95% CI, -9 to 21; p = 0.361) in PIp[o]. The 2 mAbs did not show any inhibition in sporozoite counts (Table 4).

**Table 4:** **Transmission-blocking activities of human mAb fed with infected blood meal**^a^

**Sample**	**Mean ooc**^**b**^	**PIm[o]**^**c**^	**p-value**^**d**^	**Mosquito**^**e**^	**PIp[o]**^**f**^	**p-value**^**g**^	**Mean Spz**^**h**^	**PIm[s]**^**i**^	**p-value**^**j**^
First experiment									
MAL1C	18.8	3	0.94	3/20	11	0.29	37.9	-21	0.72
	(0, 55)	(-191, 67)			(-14, 33)		(0, 94.0)	(-254, 58)	
MAL2A	7.5	61	0.09	7/20	32	0.02	39.0	-24	0.63
	(0, 37)	(-24, 87)			(7, 55)		(0.5, 89.0)	(-215, 51)	
Contl	19.5	N/A	N/A	1/20	N/A	N/A	31.2	N/A	N/A
	(0, 36)						(0, 146.5)		
Second experiment									
MAL1C	33.8	-21	0.71	0/20	0	1.000	N.D.	N.D.	N.D.
	(12, 59)	(-248, 56)			(-19, 16)				
MAL2A	42.2	-51	0.46	0/20	0	1.000	N.D.	N.D.	N.D.
	(13, 71)	(-344, 49)			(-19, 16)				
Contl	27.9	N/A	N/A	0/20	N/A	N/A	N.D.	N.D.	N.D.
	(1, 55)								

^a^All mAb were tested at 0.231 mg/ml and fed with infected blood meal at the same time.

^b^Arithmetic mean (range) of oocysts per mosquito midgut.

^c^Per cent inhibition of mean oocyst intensity (PIm[o]) and the 95% confidence interval (95% CI).

^d^Two-sided p-values for testing whether PIm[o] is significantly different from 0.

^e^Number of mosquitoes without any oocysts/number of mosquitoes examined.

^f^Per cent inhibition of prevalence of mosquitoes with oocysts (PIp[o]) and the 95% CI.

^g^p-values of PIp[o].

^h^Arithmetic mean (range) of sporozoites (×10^3^) per mosquito. No sporozoite count in the second experiment.

^i^Per cent inhibition of mean sporozoite intensity (PIm[s]) and the 95% CI.

^j^p-values of PIm[s].

Serial-feed experiments were also performed with the mAb, similar to the polyclonal antibodies, on days 3 (Table 5) or 6 (Table 6) after gametocyte feed. Neither of the mAb showed significant inhibition in oocyst intensity (PIm[o]) or oocyst prevalence (PIp[o]) in both experiments. For the inhibition in sporozoite intensity (PIm[s]), the inhibitory activity of MAL1C was not consistent (53 and -129% inhibitions in the day 3 and 6 serial feeds, respectively), and MAL2A mAb did not show any inhibition in both serial-feeds.

**Table 5:** **Transmission-blocking activities of human mAb fed three days post-infected blood meal**^a^

**Sample**	**Mean ooc**^**b**^	**PIm[o]**^**c**^	**p-value**^**d**^	**Mosquito**^**e**^	**PIp[o]**^**f**^	**p-value**^**g**^	**Mean Spz**^**h**^	**PIm[s]**^**i**^
MAL1C	15.1	2	0.97	1/20	-27	0.08	9.0	53
	(0, 36)	(-185, 65)			(-80, 3)			
MAL2A	11.6	24	0.64	1/20	-27	0.08	19.8	-4
	(0, 30)	(-119, 74)			(-80, 3)			
Contl	15.3	N/A	N/A	5/20	N/A	N/A	19.0	N/A
	(0, 44)							

^a^All mAb were tested at 0.231 mg/ml and fed three days post-infected blood meal.

^b^Arithmetic mean (range) of oocysts per mosquito midgut.

^c^Per cent inhibition of mean oocyst intensity (PIm[o]) and the 95% confidence interval (95% CI).

^d^Two-sided p-values for testing whether PIm[o] is significantly different from 0.

^e^Number of mosquitoes without any oocysts/number of mosquitoes examined.

^f^Per cent inhibition of prevalence of mosquitoes with oocysts (PIp[o]) and the 95% CI.

^g^p-values of PIp[o].

^h^Number of sporozoites (×10^3^) per mosquito calculated from a pool of salivary glands of 12 mosquitoes.

^i^Per cent inhibition of mean sporozoite intensity (PIm[s]).

**Table 6:** **Transmission-blocking activities of human mAb fed six days post-infected blood meal**^a^

**Sample**	**Mean ooc**^**b**^	**PIm[o]**^**c**^	**p-value**^**d**^	**Mosquito**^**e**^	**PIp[o]**^**f**^	**p-value**^**g**^	**Mean Spz**^**h**^	**PIm[s]**^**i**^
MAL1C	48.5	-49	0.44	2/20	10	0.15	50.3	-129
	(0, 88)	(-331, 49)			(-8, 30)			
MAL2A	42.1	-29	0.61	0/20	0	1.00	23.3	-6
	(20, 82)	(-287, 54)			(-19, 16)			
Contl	32.6	N/A	N/A	0/20	N/A	N/A	22.0	N/A
	(11, 64)							

^a^All mAb were tested at 0.231 mg/ml and fed six days post-infected blood meal.

^b^Arithmetic mean (range) of oocysts per mosquito midgut.

^c^Per cent inhibition of mean oocyst intensity (PIm[o]) and the 95% confidence interval (95% CI).

^d^Two-sided p-values for testing whether PIm[o] is significantly different from 0.

^e^Number of mosquitoes without any oocysts/number of mosquitoes examined.

^f^Per cent inhibition of prevalence of mosquitoes with oocysts (PIp[o]) and the 95% CI.

^g^p-values of PIp[o].

^h^Number of sporozoites (×10^3^) per mosquito calculated from a pool of salivary glands of 12 mosquitoes.

^i^ Per cent inhibition of mean sporozoite intensity (PIm[s]).

## Discussion

Studies have shown that anti-CS antibodies [13,29] or anti-CSPBP antibody [14] can reduce sporozoite numbers in mosquitoes. Another study has shown that the injection of CS protein or peptide into mosquitoes on day 7 or 8 post-infectious blood meal can inhibit sporozoite invasion of salivary glands [30]. It was hypothesized that anti-CS antibody induced by RTS,S/AS01 could affect oocyst formation and/or sporogony in the mosquito host. Under the conditions of this study, however, serum IgGs from RTS,S vaccinated children did not show significant inhibition in oocyst intensity, oocyst prevalence or sporozoite intensity. Similar experiments with two anti-CS human monoclonal antibodies used at over 30-fold higher levels of anti-CS specific antibodies also showed no significant reduction in oocyst intensity or prevalence, and the reduction in sporozoite intensity seen in isolated experiments was not consistent.

It is likely that for an effect of anti-CS antibodies against sporogony to occur in mosquitoes, the antibodies should be present in the right anatomical compartment at sufficient concentration. That was likely the case in past studies that demonstrated the role of CS in the physiology of sporogony [13,29]. This study suggests that the concentration of anti-CS antibodies reaching the relevant mosquito anatomic compartment following feeding on a RTS,S-vaccinated individual is too low to produce an inhibition in sporogony. Similar negative results were obtained when evaluating the effect of human anti-sporozoite antibodies induced either by natural infection [16,18] or inoculation of irradiated sporozoites [15] on the sporozoite numbers in mosquitoes. Hypothetical vaccine strategies inducing higher anti-CS antibody titres may show some inhibition, but it is unlikely considering the results of this study where anti-CS monoclonal antibodies used at high concentration failed to show any consistent inhibitory effect. Besides the antibody titre, the fine specificity could affect the results. The C-terminus and N-terminus portions of CS protein have different functions [10]. Since the RTS,S vaccine antigen does not include the N-terminal portion of CS, it was not possible to evaluate the effect of antibodies targeting the N-terminus of the protein in this study.

An alternative explanation of the negative results in this study might be linked to inherent variability in the labour-intensive biological assay, with highly variable numbers of oocysts and sporozoites within the same COM in the same feeding experiment (see [21] for discussion of this variability with respect to the oocyst counts). Indeed, the estimates of PIm[o] and PIm[s] had large 95% CIs. Other studies were also affected by this variability; 75% inhibition in PIm[s] did not reach significance (p = 0.06) in one study [31], and 90% inhibition in PIm[s] barely attained significance (p = 0.04) in another study [14]. While it would have been ideal to perform the study with more samples (e.g., test individual serum samples, instead of 6 pools) and repeat the assays many times to overcome the variability, the volumes of test materials which were available for this study were limited. In order to reduce the impact of the high variability in oocyte and sporozoite numbers, the anti-CS IgGs and control IgGs were also compared as groups. This analysis also failed to show any inhibition of sporogony.

A possible experimental limitation relates to the fact that the numbers of oocysts and sporozoites per mosquito are considerably higher in the SMFA than seen in natural infections in field-caught mosquitoes. Experiments with lower infection levels are complicated by the increased likelihood of mosquitoes in the control groups having no oocysts/sporozoites. Although unlikely in view of the results of this study, an effect under conditions of lower infectivity cannot be ruled out.

Another possible factor determining the inhibitory effect of ingested antibodies on sporogony is the timing of ingestion relative to the stage of the parasite life cycle. In past studies, researchers used different days, ranging from five to eleven days after the initial feed, to evaluate the effect [12,14-18,31]. In this study, the mosquitoes were challenged with the test antibodies three or six days after the initial gametocyte feed in the serial-feed experiments. Further investigations would be required to evaluate the effect of delaying a second feed. Relevance to mosquito behaviour in nature would need to be considered,

## Conclusions

Previous studies have clearly shown that CS protein has a significant role in sporozoite development in mosquitoes. Keeping in mind the limitations of the experimental approach (oocyst and sporozoite count variability, limited sample volume available for testing, high infectivity compared to nature, serial feeding to day 6 only), this study does not support the concept that antibodies induced by the RTS,S/AS01 vaccine in human noticeably reduce malaria transmission by blocking sporozoite development or salivary gland invasion in mosquitoes when taken up during feeding. Whether RTS,S/AS01 vaccine may reduce transmission in a population through the ability of pre-erythrocytic immunity to prevent new infections remains to be evaluated.

## Abbreviations

CS: Circumsporozoite; CSPBP: Circumsporozoite protein-binding protein; SMFA: Standard membrane feeding assay; CI: Confidence interval; mAb: Monoclonal antibody; EU: ELISA units; PBS: Phosphate buffered saline; PIm[o]: % inhibition of mean oocyst intensity; PIp[o]: % inhibition of oocyst prevalence; PIm[s]: % inhibition of mean sporozoite intensity; COM: Container of mosquitoes.

## Competing interests

Johan Vekemans and Erik Jongert are GSK employees and own GSK shares.

## Authors’ contributions

KM, EJ, CAL and JV designed the study and drafted the manuscript. JPL and CJD designed the study and conducted the human RTS,S vaccine trial. KM, BD and LZ performed the study. MPF analysed the data and drafted the manuscript. All authors read and approved the final manuscript.
